# Dietary supplementation of Astragalus polysaccharide or its nanoparticles enhances testicular hemodynamics, echotexture, scrotal circumference, concentration of testosterone, estradiol, nitric oxide, and total antioxidant capacity, and semen quality in mature Ossimi rams

**DOI:** 10.1186/s12917-025-04477-6

**Published:** 2025-02-06

**Authors:** Eman Fayez, Ali Salama, Mohamed Ahmed Ismail, Fady Sayed Youssef, Zaher Mohamed Rawash, MR Oshba, Haney Samir

**Affiliations:** 1 Department of Theriogenology, Faculty of Veterinary Medicine, Cairo University, Giza, 12211 Egypt; 2 Pharmacology Department, Faculty of Veterinary Medicine, Cairo University, Giza, Egypt; 3 Artificial Insemination and Embryo Transfer Department, Animal Reproduction Research Institute (ARRI), Agriculture Research Centre (ARC), Giza, Egypt; 4 Diagnostic Imaging and Endoscopy Unit (DIEU), Animal Reproduction Research Institute (ARRI), Agriculture Research Centre (ARC), Giza, Egypt

**Keywords:** Astragalus polysaccharide, Ram, Testicular hemodynamics, Echotexture, Semen quality, Nitric oxide, Testosterone, Estradiol, Total antioxidant capacity

## Abstract

This study investigated, for the first time, the potential role of Astragalus polysaccharide (APS) and APS nanoparticles on testicular blood flow (TBF) and semen quality in Ossimi rams. Fifteen sexually mature Ossimi rams were allocated randomly into two treated groups that orally administered either nano APS (2 g/ram/day; *n* = 5) or APS (20 g/ram/day; *n* = 5) for four weeks and a control group (*n* = 5). The nano-emulsion was prepared by adding corn oil to the APS solution, sonicated, centrifuged at 20,000 rpm, then washed 3–4 times, and vacuum dried overnight at 40 °C. The antioxidant activity of APS and APS nano-emulsion was evaluated in vitro. Blood collection and ultrasonographic assessment of the testes and supratesticular arteries (STAs) were conducted immediately before treatment (W0) and once weekly for 6 successive weeks after APS and nano APS administration (W1-W6). Serum testosterone (T) and estradiol (E_2_) concentrations were determined by ELISA kits, while nitric oxide (NO) and total antioxidant capacity (TAC) were measured spectrophotometrically. Moreover, semen collection and evaluation of some sperm parameters were performed once a week. Results revealed decreases (*P* < 0.05) in the Doppler indices (resistive index; RI, pulsatility index; PI, and systolic/diastolic; S/D) of the testicular arteries at most time points of the study in the nano APS and APS groups. Pixel intensity (PIX) and integrated density (IND) of testicular parenchyma were significantly reduced (P ˂ 0.05) in the treated groups compared to the control one. T, E_2_, NO, and TAC concentrations increased (*P* < 0.05) in the treated groups compared to the control one. Increases (*P* < 0.05) were noticed in the mass motility, progressive motility %, live sperm %, and membrane integrity % in nano APS and APS groups, compared to the control. Rams in the nano APS group had significantly higher (*P* < 0.05) sperm cell concentration than the control one. In conclusion, this study extrapolated that the dietary administration of APS and its nanoparticles can improve TBF, testicular echotexture, sperm characteristics, and the concentration of serum T, E_2_, NO, and TAC with a more significant effect in the APS nanoparticles compared with APS. So, it could be recommended as a dietary supplementation (2 g/ram/day) for enhancing the reproductive performance of rams.

**Supplementary Information:**

The online version contains supplementary material available at 10.1186/s12917-025-04477-6.

## Introduction

Nowadays, as the world population grows and food supplies become scarce, it is vital to enhance food animal production to provide a sustainable source of meat, milk, and other animal products. This occurs by improving the reproductive capacity of livestock animals. Among livestock animals, sheep stand out for their ability to produce meat, milk, lamb, and wool under adverse environmental circumstances [1]. Reproduction is one of the most significant economic variables in the livestock industry, and it is determined not only by the reproductive potential of the female but also by the male.

The testicles have a high metabolic rate that facilitates both spermatogenesis and steroidogenesis. Oxygen, nutrients, and different hormones are primarily transported to and from the testis by the testicular blood vessels [2, 3]. Maintaining a sufficient arterial blood supply is essential because testicular blood flow (TBF) has a significant impact on testicular function. TBF variations are typically accompanied by significant variations in seminal and plasma testosterone concentrations, as well as sperm production in men [4, 5], bulls [6, 7], rams [8, 9], and stallions [10, 11]. Studies have also demonstrated the significance of TBF in assessing male reproductive capacity and diagnosing a range of reproductive disorders. Incorporating eligible rams into breeding programs and determining the TBF in testicular arteries can both be aided by color Doppler ultrasonography [12]. Furthermore, several Doppler measures are believed to be possible markers of seminal quality, such as the pulsatility index (PI) and resistive index (RI) of the testicular artery [13].

Developing highly productive breeds requires an understanding of the morphometric testis dimensions and spermatological traits that determine a ram’s fertility. Early testicular morphometric measurements offer a selective advantage for rams intended for artificial insemination or breeding. The amount of sperm and the volume of the ejaculate in rams are greatly influenced by testicular development. According to Belkhiri et al. [14] males with larger testicles have the capacity to generate more sperm than those with smaller testicles. Assessing the size, shape, and the echotexture of testes is part of the morphometric evaluation process of fertility potentials in small ruminants [12].

Astragalus polysaccharide (APS) is a bioactive water-soluble heteropolysaccharide isolated from the stems or dried roots of *Astragalus membranaceus*. *Astragalus membranaceus* is one of the most popular herbal medicines worldwide. It is known in China as “Huangqi.” The most major natural active component in *Astragalus membranaceus* is APS, which has numerous pharmacological actions [15] because of its low toxicity, low residue, and tolerance thresholds [16]. Moreover, APS has been claimed to have antibacterial and antiviral characteristics and blood glucose regulation and can be utilized as an immunity booster [16, 17]. APS can be classified as a good antioxidant agent as proposed by several reports. APS could prevent mitochondrial damage induced by oxidative stress in mice by scavenging ROS [18]. In farm animals, several studies revealed the positive effects of APS administration on male fertility. In goats, APS significantly improved the sperm quality and antioxidant capacity of frozen-thawed spermatozoa [19]. Furthermore, when APS is added to frozen boar sperm, it can boost the sperm’s antioxidant ability and enhance the parameters of in vitro fertilization (IVF) as well as the outcomes of embryonic development [17]. It could also dramatically enhance the motility and survivability of human spermatozoa in vitro [20] and considerably increase sperm kinematics, acrosome integrity, plasma membrane integrity, and mitochondrial function of bull [21]. Till now, there are no studies investigating the efficacy of dietary supplementation of APS on the fertility potential of rams.

Nanotechnology is a rapidly developing branch of biotechnology, with potential biomedical uses emerging in recent years. Nanoparticles are particles with at least one dimension between 1 and 100 nm [22]. Due to their physicochemical characteristics, nanoparticles can encapsulate a significant amount of drug, protecting it from degradation, and increasing its bioavailability, stability, and pharmacokinetic properties, while reducing possible toxic side effects [23]. Nanoscale structures show functional features that are special and not seen at the macroscale . This is due to the new properties that the nanoscale particles display, such as a specific surface area, multiple active surface centers, high surface activity, high catalytic efficacy, and potent adsorption capacity [24, 25].

As far as we are aware, there hasn’t been any research on the impact of APS and nano APS on testicular hemodynamics along with testicular echotexture, scrotal circumference, concentration of testosterone (T), estradiol (E_2_), nitric oxide (NO), total antioxidant capacity (TAC), and semen analysis in mature rams. APS acts as an antioxidant and prevents mitochondrial damage induced by oxidative stress. Therefore, we hypothesized that the dietary administration of APS or its nanoparticles could improve ram reproductive performance through its stimulating effects on testicular echogenicity, hemodynamics, and semen parameters. We aimed to determine the impact of oral administration of APS and nano-APS on testicular hemodynamics, echotexture, steroid production, antioxidant status, and semen parameters of mature rams.

## Materials and methods

This study was carried out at the educational farm, Faculty of Veterinary Medicine, Cairo University, Giza, Egypt, and at the Artificial Insemination Department, Animal Reproduction Research Institute, Agricultural Research Centre at Giza. All experimental procedures were accepted by the Ethics Committee for Animal Use at the Faculty of Veterinary Medicine, Cairo University, Egypt (approval number: Vet CU 25122023867).

### Preparation of Astragalus membranaceus root powder extract and its nanoparticles

#### Chemicals and reagents

All chemicals and reagents used in these procedures, unless otherwise noted, were purchased from Sigma, St. Louis, MO, USA.

#### Extraction process

The extraction of water-soluble polysaccharides was conducted according to the protocol of Yang et al. [26]. The dried roots of *Astragalus membranaceus* (100 g) were crushed and subjected to extraction with 2 L of deionized water at 60 °C for 2 h. After filtration and centrifugation at 9000 g for 20 min, the supernatants were collected and concentrated at 60 °C by a vacuum rotary evaporator (Eyela N-1100 V‐W, Tokyo Rikakikai Co. Ltd., Tokyo, Japan). Anhydrate ethanol was added to a final concentration of 60% (v/v) and incubated for 12 h at 4 °C in a refrigerator. The pellets were collected by centrifugation at 6000 g for 20 min, and freeze‐dried to obtain the water‐soluble polysaccharides (APS). A simple water extract of *Astragalus membranaceus* was evaporated to a predetermined amount, then ethanol (> 99% purity) was added to allow the mixture to stand overnight. After that, the resultant extract was separated and centrifuged. Using traditional phytochemical methods, alkaloids, tannins, flavonoids, carbohydrate/ glycosides, and resin were all determined in *Astragalus membranaceus* extract according to the method described by Pant et al. [27].

#### Aqueous phase preparation

APS (80 mg) dissolved in 20 mL of deionized water to give an APS solution of 4 mg/mL. In the APS group, APS was dissolved in deionized water at a concentration of 580 mg/mL. Surfactant (anionic sodium dodecyl sulfate, SDS) was added dropwise under magnetic stirring at 500 rpm for 30 min to obtain a stable aqueous phase.

#### Nano-Emulsion preparation and characterization

In the nano-APS group, APS was dissolved in APS solution at a concentration of 58 mg/mL. Oil: nano-APS 1:10 volume of oil and deionized water. The oil and aqueous APS solutions were then mixed with vigorous magnetic stirring at 500 rpm for 15 min.

#### Ultrasonication

The resultant coarse emulsion was further sonicated using an ultrasonic probe system at 60% amplitude with a 1-second on-off pulse. Total sonication time varied from 5 to 15 min to determine optimum nano-emulsion droplet sizes [28].

#### Isolation and drying

The prepared APS nano-emulsion was centrifuged at 20,000 rpm to sediment the APS nanoparticles formed. The nanoparticles were washed 3–4 times and finally vacuum dried overnight at 40 °C.

#### Method of characterization

We have characterized APS nano-emulsion to determine its physical and chemical properties. To determine the shape and surface topography of APS nano-emulsion, transmission electron microscopy (TEM; model of Jeol, JEM-2100 high-resolution, Japan) and an atomic force microscope (AFM, Agilent, USA) were used. A dynamic light scattering experiment was performed to determine how well particles dispersed in fluids based on their zeta potential and size.

#### Determination of antioxidant activity of APS and APS nano-emulsion in vitro

To evaluate the free radical scavenging abilities of APS and APS nano-emulsion compared to regular vitamin C [29], 1,1-Diphenyl-2-picrylhydrazyl (DPPH) radical-scavenging assay (DPPH assay) was used as shown in Fig. 2. We centrifuged 1.0 mL of APS and APS nano-emulsion at different concentrations (100, 50, 25, 12.5, 6.25, 3.12, 1.56 g/mL) with 1.0 mL of DPPH (1.0 M in methanol), followed by 30 min incubation at room temperature. To measure the absorbance at 517 nm, a UV-VIS spectrophotometer was used (Systronics, AU-2701). We also tested the sample with DPPH and chemicals that were not in the sample as a control. We used a methanol blank solution. As a measure of free radical scavenging activity, the following formula was used: Percentage of scavenging = (Pc − Ps)/Pc × 100. Where Pc = absorbance of vitamin C and Ps = APS and APS nano-emulsion.

#### Particle size and zeta potential analysis

The particle size and surface charge of synthesized APS nano-emulsion were characterized using a Nanotrac-Wave II Zeta sizer (Microtrac, USA).

### Animals

Fifteen sexually mature Ossimi rams (Ovis aries) aged 1–3 years old and weighing 40–70 kg (54.33 ± 2.51 kg) were used in the present study in the winter of 2024 (from late December to early March) according to the Egypt Metrological Agency. Ossimi rams are fat-tailed Egyptian sheep reared primarily for meat and wool production. This breed has a limited reproductive capacity during the summer months (from June to early September) [30]. Clinical, andrological, and ultrasonographic exams showed that they were clinically healthy and had no cardiovascular or reproductive problems. All the rams were housed in a ram pen (32 square meters) with an open front and a concrete floor. Dry feeders with one drinker were provided in the ram pen. They were fed rations according to NRC-2007 [31] recommendations constituting 400 g of pelleted concentrate/ram/day (Supplement Table 1) and 850 g of chopped hay and green forage. Animals had *ad libitum* access to fresh water and salt licks. The rams were routinely vaccinated and treated for external and internal parasites.

### Experimental design

**Fig. 1:**
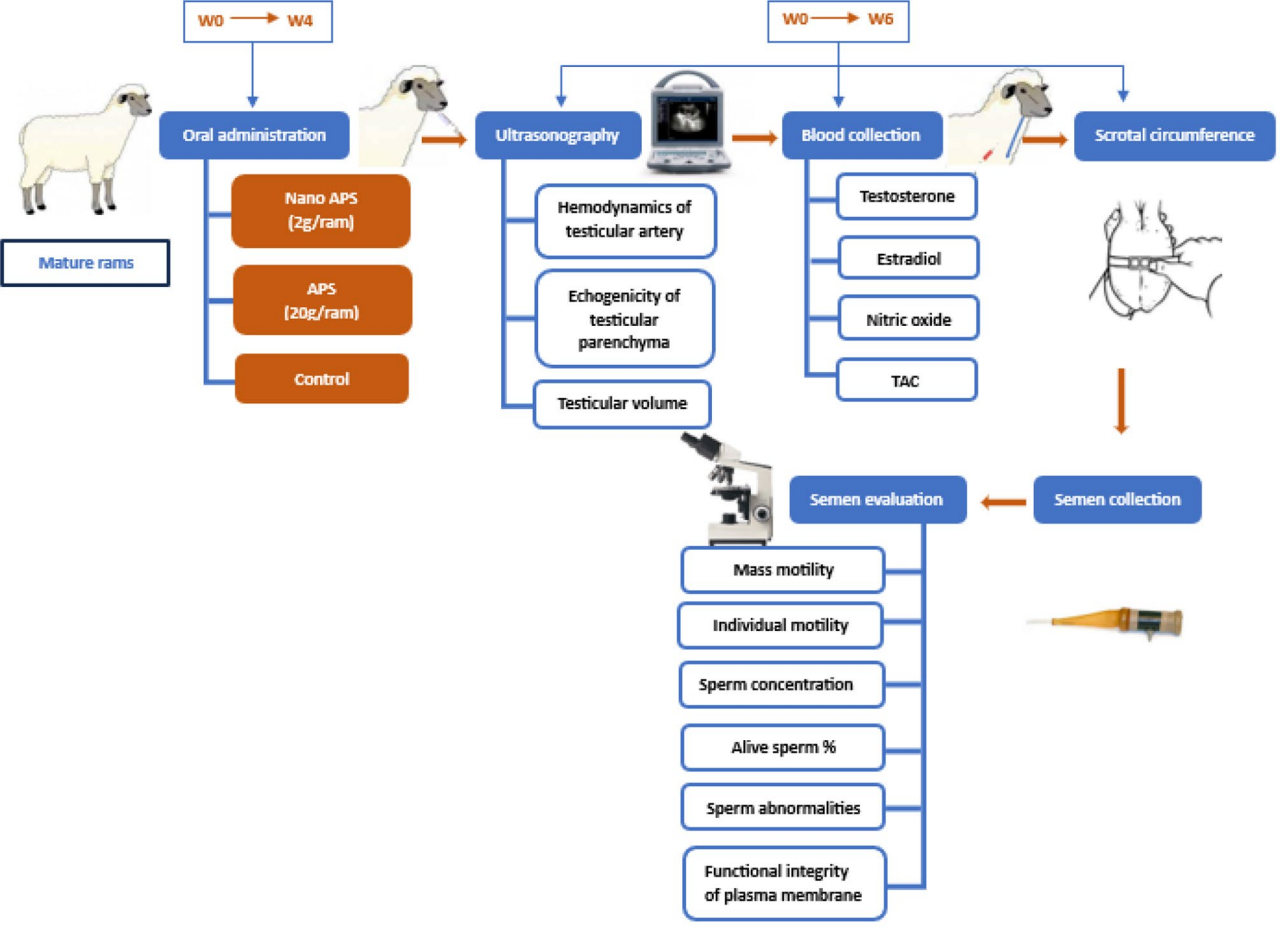
Schematic illustrations of the experimental procedures in this study

An overview of the experimental design is given in Fig. 1. Rams were randomly allocated into three equal groups (*n* = 5, each): (1) nano APS group was orally administered nano APS (2 g/ram/day), (2) APS group was orally administered APS (20 g/ram/day) for four weeks (after 2 weeks of adaptation) as previously stated [32], and (3) a control group that received neither APS nor nano APS (a placebo; water). Ultrasound examination of the right and left testes, blood sampling, and semen collection and evaluation were performed once a week starting from the first day of administration (W0) and for 6 consecutive weeks (W1-W6) to cover the spermatogenesis [33]. The dose used for the nano-emulsion of APS in our study (10 times less than the APS) was selected as previously recommended [34].

### Ultrasonographic examinations

Ultrasound assessments were applied by the same operator using a Doppler scanner equipped with a 7.5 MHz linear-array probe (EXAGO, IMV, France). The rams were gently restrained without being sedated. Before each ultrasonographic test, fine wool was shaved from both sides of the scrotum. A substantial amount of scanning gel was used to ensure contact and reduce scanning artifacts. All ultrasound machine parameters (frequency, brightness, depth, and contrast) were adjusted and fixed for all examinations. Hemodynamic changes in testicular arteries were evaluated in this study. The testicular artery of rams appears convoluted just before entering the testis. The convoluted segment is called the supratesticular artery (STA) [35]. For pulsed Doppler evaluations, the transducer was placed vertically on the side of the scrotum and then moved dorsally until the STA appeared inside the vascular network in the testis’ proximal pole as previously illustrated [36]. After assessing the spectral pattern of the right and left STAs, the resistance index (RI) and pulsatility index (PI) were determined [37].

Blood perfusion of tissue downstream exhibited an inverse relationship with an artery’s RI and PI values [38]; consequently, as RI and PI increase, vascular resistance to blood flow increases, lowering blood perfusion, and vice versa [36]. Each parameter was calculated using two to four measurements obtained at sites of interest along the STA [39]. To assess testicular echogenicity, a clean, artifact-free image of both testes was captured using B-mode ultrasonography, frozen, and saved for later computed analysis. Using Adobe Photoshop CC software, the saved photos were recovered and examined for testicular echogenicity by placing a 1 cm × 1 cm square in the testicular parenchyma in three unique locations respecting the central positioning of the mediastinum testis [40]. An ultrasonogram is a matrix of pixels whose values vary from 0 (anechoic black) to 255 (absolute white) [41]. The testicular parenchyma was measured for length (L), width (W), and thickness (T) using electronic calipers. The formula for determining testicular volume (TV) is L × W × T × 0.61 [42].

### Scrotal circumference

The scrotal circumference of the animals was measured (cm) at the greatest width of the testicle with a flexible measuring tape [43].

### Blood sampling

Blood samples (3 ml) were obtained from each ram through jugular venipuncture into plain tubes just before ultrasonographic assessments. The blood samples were centrifuged for 10 min at 3000 rpm, after which the serum samples were collected and kept at -20 °C until hormonal and biochemical analyses were performed.

### Hormonal analysis

Concentrations of T (ng/ml) and E_2_ (pg/ml) were analyzed using the commercial ELISA kits (DiaSino Laboratories Co., Ltd. Zhengzhou, China) as described by the manufacturers. The intra- and inter-assay coefficients of variation, respectively, were 3.3 and 4.8% for testosterone and 3.8 and 5.6% for estradiol. Assay sensitivity was 0.05 ng/ml for testosterone and 20 pg/ml for estradiol.

### Biochemical analysis

Concentrations of NO (µmol/L) and TAC (mM/L) were measured using commercial kits (Bio-diagnostic, Giza, Egypt) spectrophotometrically (Prietest TOUCH, Robonic, India) following the manufacturer’s instruction at a wavelength of 540 nm and 505 nm respectively. The NO intra-assay coefficient variation was 5.3%, with an assay sensitivity of 0.225 µmol/l in nitrite form. The TAC intra-assay coefficient was 3.4% and 0.04 mM/L sensitivity.

### Semen collection and evaluation

All rams were trained for semen collection using teaser ewes and an artificial vagina for 1.5 months before the start of the study. In this investigation, two ejaculates were collected from each ram in the early morning using an artificial vagina set at 41–42 °C with lubrication once a week during the study (W0-W6). Ejaculates were taken to the laboratory and put in a 30 °C warm water bath immediately after collection. The following standard laboratory procedures were used to evaluate semen samples.

#### Mass motility

The mass motility of the spermatozoa was estimated according to Evans and Maxwell [44]. A drop of semen sample was placed on a clean, warm dry glass slide, viewed under the microscope (LABOMED, Labo America, Inc., U.S.A) at low magnification, and scored on a scale from 0 (no motility) to 5 (excellent motility) according to Moule [45].

#### Individual motility

Individual motility (%) was investigated microscopically (400x) using an adjusted hot stage at 38–40 °C in semen samples diluted with 2.9% sodium citrate dihydrate solution and placed on a glass slide and evenly under a glass cover slide. After inspecting multiple microscopic fields, the individual sperm motility percent was assessed on a subjective range of 0- 100% to the closest 5% [46].

#### Sperm concentration

Sperm concentration (x10^9^ sperm/ml) was estimated as described by Evans and Maxwell [44] using a hemocytometer (Neubauer counting chamber) and examined under a microscope (400x).

#### Alive and abnormal sperm percentages

The alive sperm percentage was determined using an eosin-nigrosin stain reported by Campbell et al. [47]. On a warmed glass slide, combine 2 µl semen and 10 µl eosin-nigrosin stain (1.7 gm Eosin, 10 gm Nigrosin, and 2.9 gm sodium citrate in 100 ml distilled water). A total of 200 sperm cells were evaluated using a 400x magnification objective microscope. Alive spermatozoa avoid the eosin stain and appear white, but dead spermatozoa take the stain and appear reddish. The evaluation of total sperm abnormalities was carried out on the same slide used for the assessment of live sperm. Two hundred sperm cells were examined with a microscope under an oil immersion lens (1000x). Total sperm abnormalities were recorded.

#### Functional integrity of plasma membrane

The functional membrane integrity of ram sperm was determined using a hypo-osmotic swelling test (HOST) reported by Revell and Mrode [48]. In an Eppendorf tube, 10 µl semen was diluted with 100 µl hypo-osmotic solution (4.9 g sodium citrate, 9 g fructose in 1000 ml distilled water) and incubated at 37^o^ C for 45 min. Following incubation, a tiny drop of the mixture (5 µl) was put on a microscope slide, coverslipped, and immediately examined by a bright-field microscope (400× magnification). Spermatozoa with functional plasma membranes appeared swollen with coiled tails, while inactive spermatozoa remained unchanged. Sums of 200 spermatozoa in duplicate smears were examined in five different microscopic fields. The number of sperm that appeared swollen with coiled tails was recorded.

### Statistical analysis

To ascertain the homogeneity and type of data, the normalcy test was performed on the data using the Kolmogorov-Smirnov test. Since the right and left testes of the rams under investigation in this study did not show any significant differences [49, 50], the data for each ram was combined and compared between the groups. All data were normally distributed and presented as means ± standard error of the mean (SEM) for the studied parameters. To examine the impact of treatment as a fixed factor and time as a repeating factor, all parameters were compared for differences using repeated measures of two-way ANOVA. Assessment was done at different time points using the Bonferroni post hoc test to determine the impact of treatment (3 levels: APS versus nano APS versus control) on changes in TBF in the STA, testicular echogenicity, testicular volume, scrotal circumference, concentrations of T, E_2_, NO, and TAC, and semen quality parameters. The software GraphPad Prism5 was used for all statistical analyses. Significance was defined as *P* < 0.05.

## Results

### Characterization of APS nano-emulsion

**Fig. 2:**
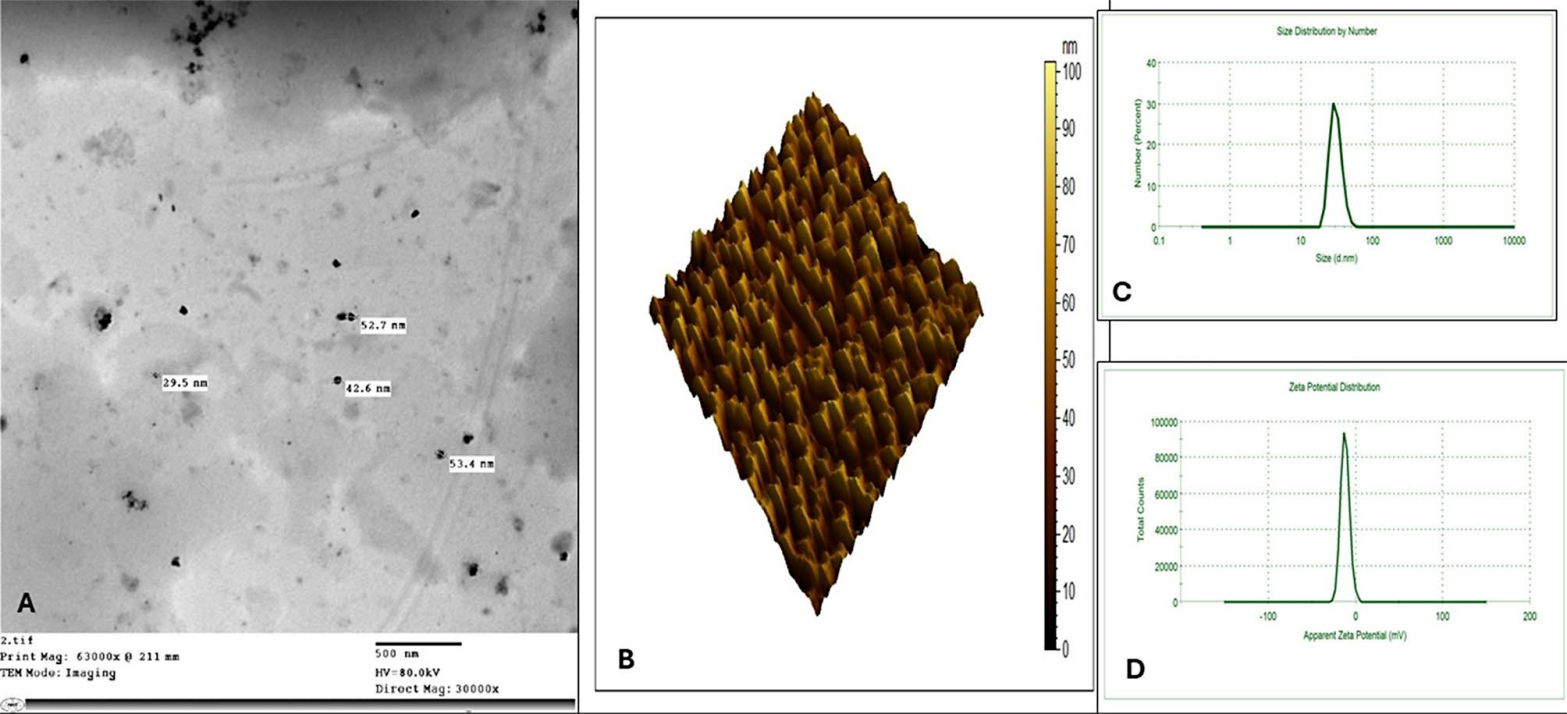
Results of characterization techniques of nanoemulsions containing Astragalus Polysaccharides (APS) shown under a transmission electron microscope as spherical particles with a diameter of 20–50 nm and a maximum thickness of 20–50 nm (**A**). APS nano-emulsion does not tend to aggregate in some regions with a homogeneous dispersed matrix (**B**). The results of zeta size showed that the average particle size of synthesized APS nano-emulsion is 59.10 ± 0.20 nm (**C**). Results of zeta potential showed + 31.2 ± 0.01 mV (**D**)

The results of characterization techniques are shown in Fig. 1. According to TEM images of the APS nano-emulsion, the resulting particles are spherical to subspherical objects with a diameter of 20–50 nm and a maximum thickness of 20–50 nm (Fig. 2A). Furthermore, it has been found that APS nano-emulsion does not tend to aggregate in some regions with homogeneous dispersed matrix (Fig. 2B). The results of zeta size showed that the average particle size of synthesized APS nano-emulsion is 59.10 ± 0.20 nm (Fig. 2C). Results of zeta potential showed + 31.2 ± 0.01 mV as shown in Fig. 2D. Accordingly, the high zeta potential of the synthesized nano-emulsion directly impacts colloidal stability in water, as it derives from the high bioactivity of the APS nano-emulsion.

### Antioxidant activity of APS and APS nano-emulsion in vitro

**Fig. 3:**
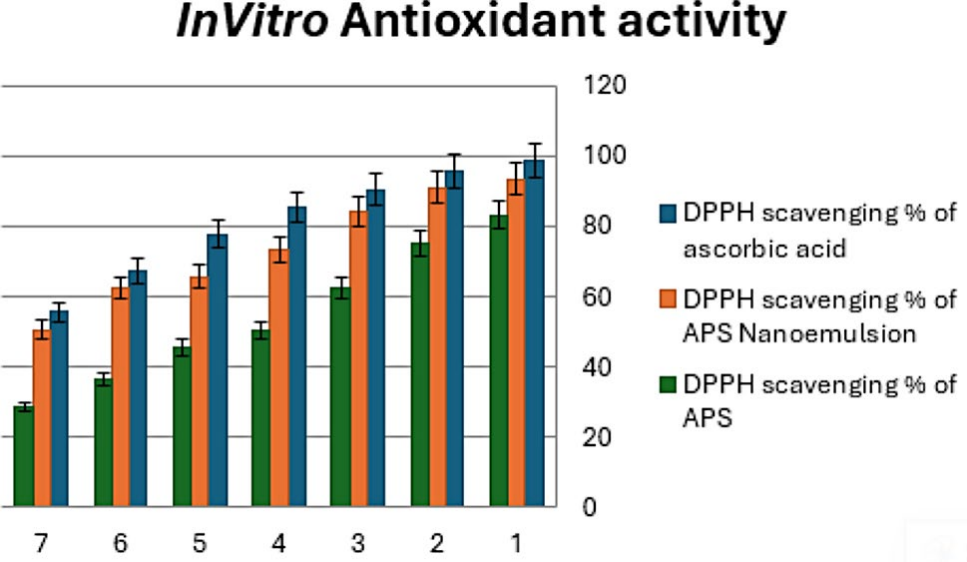
Differences between groups in the % of free radical scavenging activity of Astragalus Polysaccharides (APS) and APS nano-emulsion against ascorbic acid

Results for the DPPH radical scavenging method used to test the antioxidant activity of APS and APS nano-emulsion at the various tested concentrations (100, 50, 25, 12.50, 6.25, 3.12, 1.56 g/mL) represented as 1, 2, 3, 4, 5, 6, and 7 (Fig. 3). APS nano-emulsion showed antioxidant activity and the percentage of antioxidant activity increased in a dose-dependent manner that was comparable to that of normal ascorbic acid and APS. *Broccoli (Brassica oleracea var. italica)* was found to have considerable DPPH radical scavenging abilities as represented in Fig. 3.

### Testicular hemodynamics

**Fig. 4:**
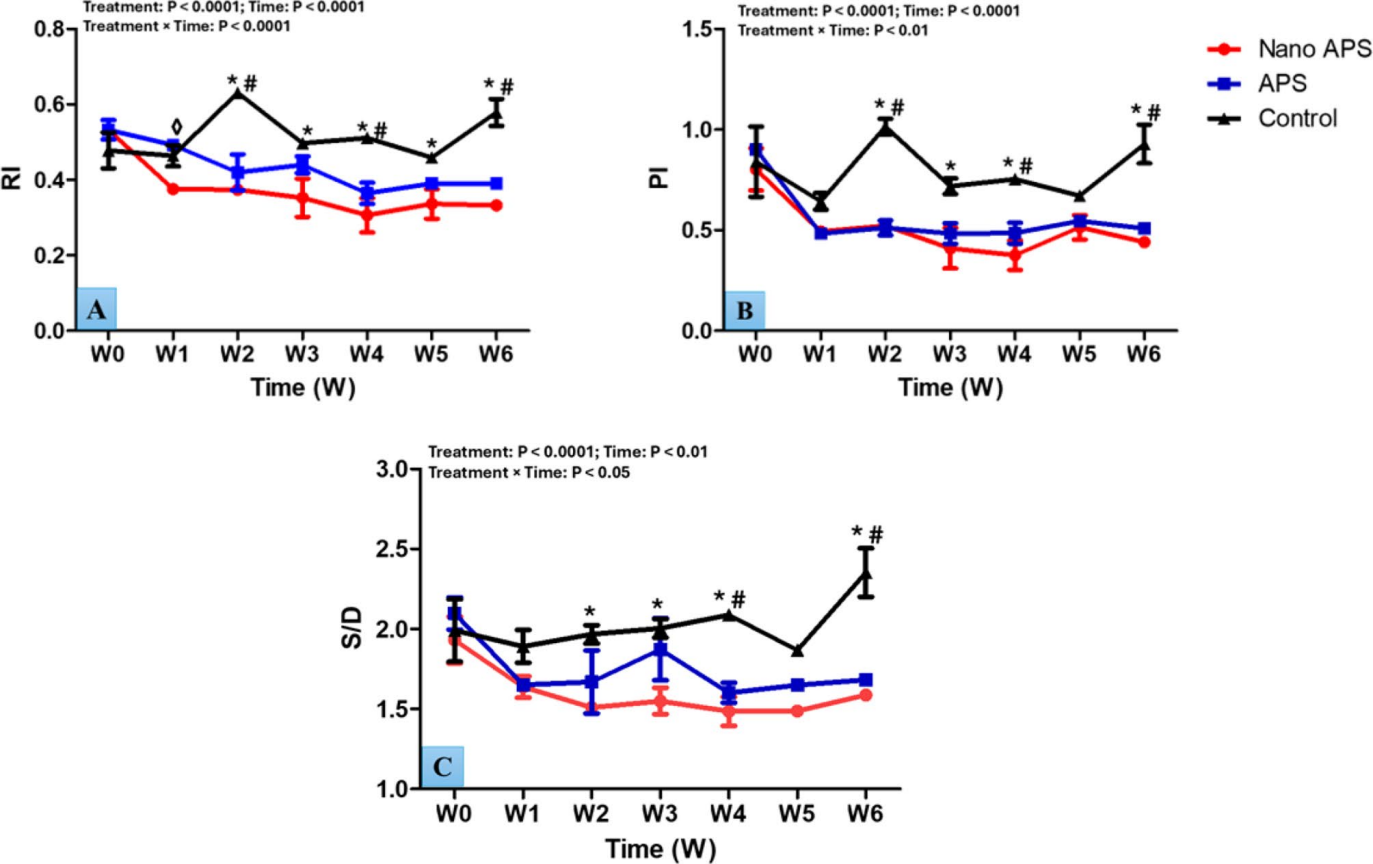
Changes in the parameters of testicular hemodynamics [resistive index; RI (**A**), pulsatility index; PI (**B**), and systolic/diastolic; S/D (**C**)] at the level of supratesticular artery as measured by pulsed-wave Doppler ultrasonography in Ossimi rams in the nano APS group, APS group, and the control group (*n* = 5, each) during different times (weeks). Values are means ± SEM. ◊ Values represent significant differences (*P* < 0.05) between nano APS and APS groups at the indicated times during the study. * Values represent significant (*P* < 0.05) differences between nano APS and control groups during the study. # Values represent significant (*P* < 0.05) differences between APS and control groups during the study

Referring to the experimental groups in this study, the changes in the parameters of testicular hemodynamics during the different times (weeks) are presented in Fig. 4. The significance showed that the decrease in RI values depends on APS type (treatment) and time, and their interaction (*P* < 0.0001, for all). RI values of blood flow were significantly lower (*P* < 0.05) in the nano APS group compared to the APS one, specifically at W1 (0.38 ± 0.01 and 0.49 ± 0.003; respectively). Compared to the control group, the nano APS group attained lower (*P* < 0.05) RI values specifically at the time point from W2 to W6. Furthermore, the APS group yielded lower (*P* < 0.05) RI values at W2, W4, and W6 than the control one. Treatment, time, and interaction were significantly different (*P* < 0.01, *P* < 0.0001, and *P* < 0.0001; respectively) in values of PI of TBF. On the other hand, decreases (*P* < 0.05) in the values of PI were observed in the nano APS group compared to that in the control one at most time points (W2, W3, and W4) of the study. Rams in the APS group showed lower (*P* < 0.01) PI values, especially at W2, W4, and W6 compared to the control one. Furthermore, there were treatment, time, and interaction effects on values of systolic/diastolic (S/D) (*P* < 0.0001, *P* < 0.01, and *P* < 0.05; respectively ) of TBF. Values of S/D decreased (*P* < 0.05) in the nano APS administrated group at most time points in the study (W2, W3, W4, and W6) compared to those in the control one. Rams in the APS administrated group attained lower (*P* < 0.01) S/D values at W4 and W6 than in the control.

### Testicular echogenicity

**Fig. 5:**
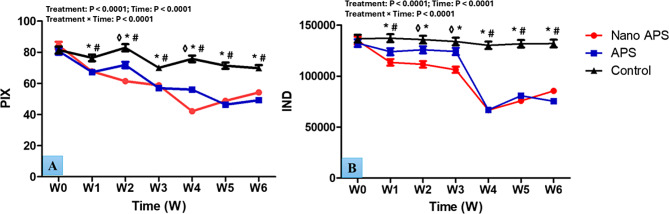
Changes in echotexture of testicular parenchyma [pixel intensity; PIX (**A**) and integrated density; ID (**B**)] as measured by computer analysis software in Ossimi rams in the nano APS group, APS group, and the control group (*n* = 5, each) during different times (weeks). Values are means ± SEM. ◊ Values represent significant differences (*P* < 0.05) between nano APS and APS groups at the indicated times during the study. * Values represent significant (*P* < 0.05) differences between nano APS and control groups during the study. # Values represent significant (*P* < 0.05) differences between APS and control groups during the study

The changes in echogenicity of the testicular parenchyma in the studied groups are shown in Fig. 5. In general, treatment, time, and interaction had notable differences (*P* < 0.0001, for all) in values of pixel intensity (PIX) and integrated density (IND) of testicular parenchyma. There were significant (*P* < 0.001) decreases in the values of PIX of the testicular parenchyma in the nano APS group, especially at W2 and W4 compared to the APS one. Values of PIX decreased (*P* < 0.01 and *P* < 0.001) in the nano APS and APS groups at the time point from W1 to W6, compared to its values in the control one. Rams in the nano APS group had lesser (*P* < 0.05) IND values at W2 and W3 than those in the APS group. The APS group attained lower (*P* < 0.001) IND values from W1 to W6, compared to the control one. Values of IND in the APS group were lower at most time points (W1, W4, W5, and W6) in the study, compared to that in the control one.

### Testicular volume and scrotal circumference

**Fig. 6:**
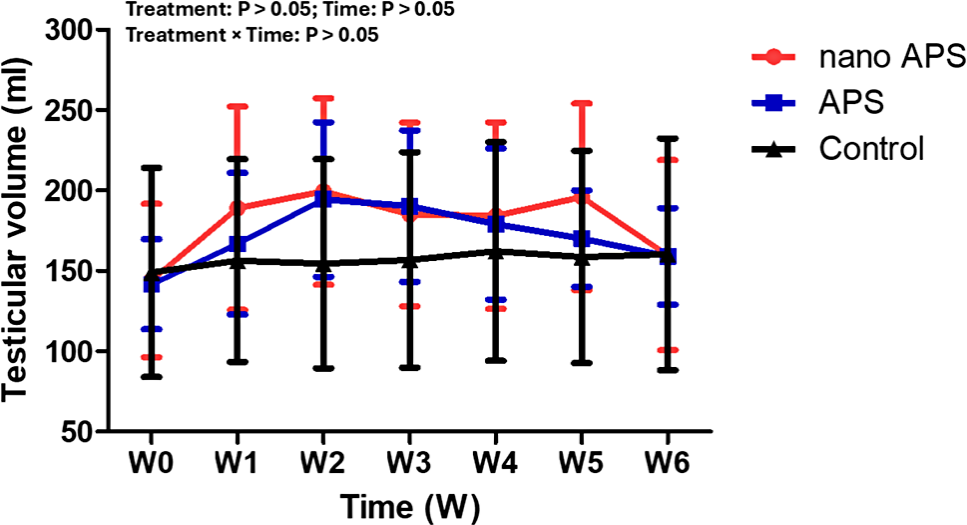
Changes in testicular volume (ml) in Ossimi rams in the nano APS group, APS group, and the control group (*n* = 5, each) during different times (weeks). Values are means ± SEM. ◊ Values represent significant differences (*P* < 0.05) between nano APS and APS groups at the indicated times during the study. * Values represent significant (*P* < 0.05) differences between nano APS and control groups during the study. # Values represent significant (*P* < 0.05) differences between APS and control groups during the study

**Fig. 7:**
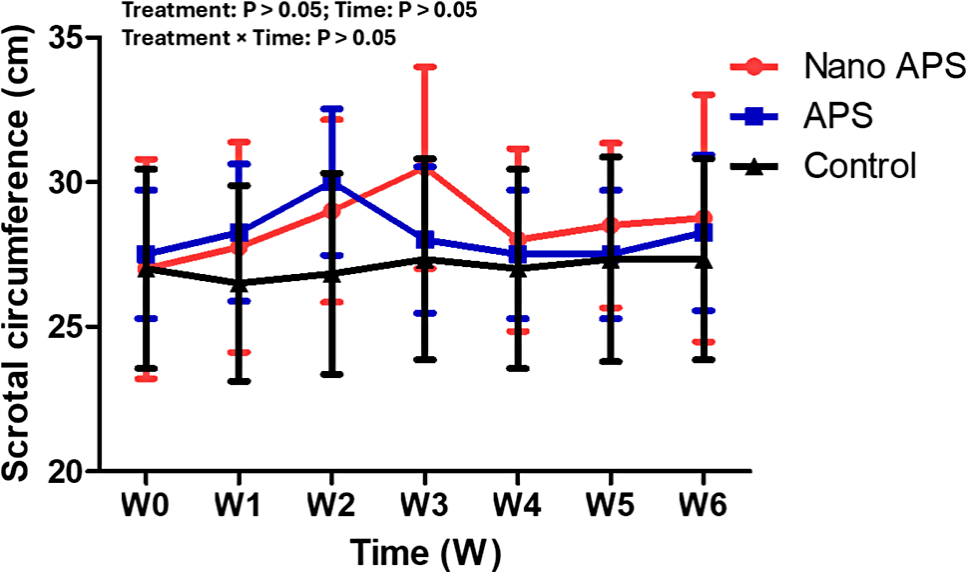
Changes in scrotal circumference (cm) in Ossimi rams in the nano APS group, APS group, and the control group (*n* = 5, each) during different times (weeks). Values are means ± SEM. ◊ Values represent significant differences (*P* < 0.05) between nano APS and APS groups at the indicated times during the study. * Values represent significant (*P* < 0.05) differences between nano APS and control groups during the study. # Values represent significant (*P* < 0.05) differences between APS and control groups during the study

The effect of nano APS and APS administration on testicular volume (ml) and scrotal circumference (cm) in the present study is presented in Figs. 6 and 7 respectively. The values for testicular volume and scrotal circumference in the nano APS and APS groups compared with the control group were not significant (*P* > 0.05).

### Hormonal and biochemical analysis

**Fig. 8:**
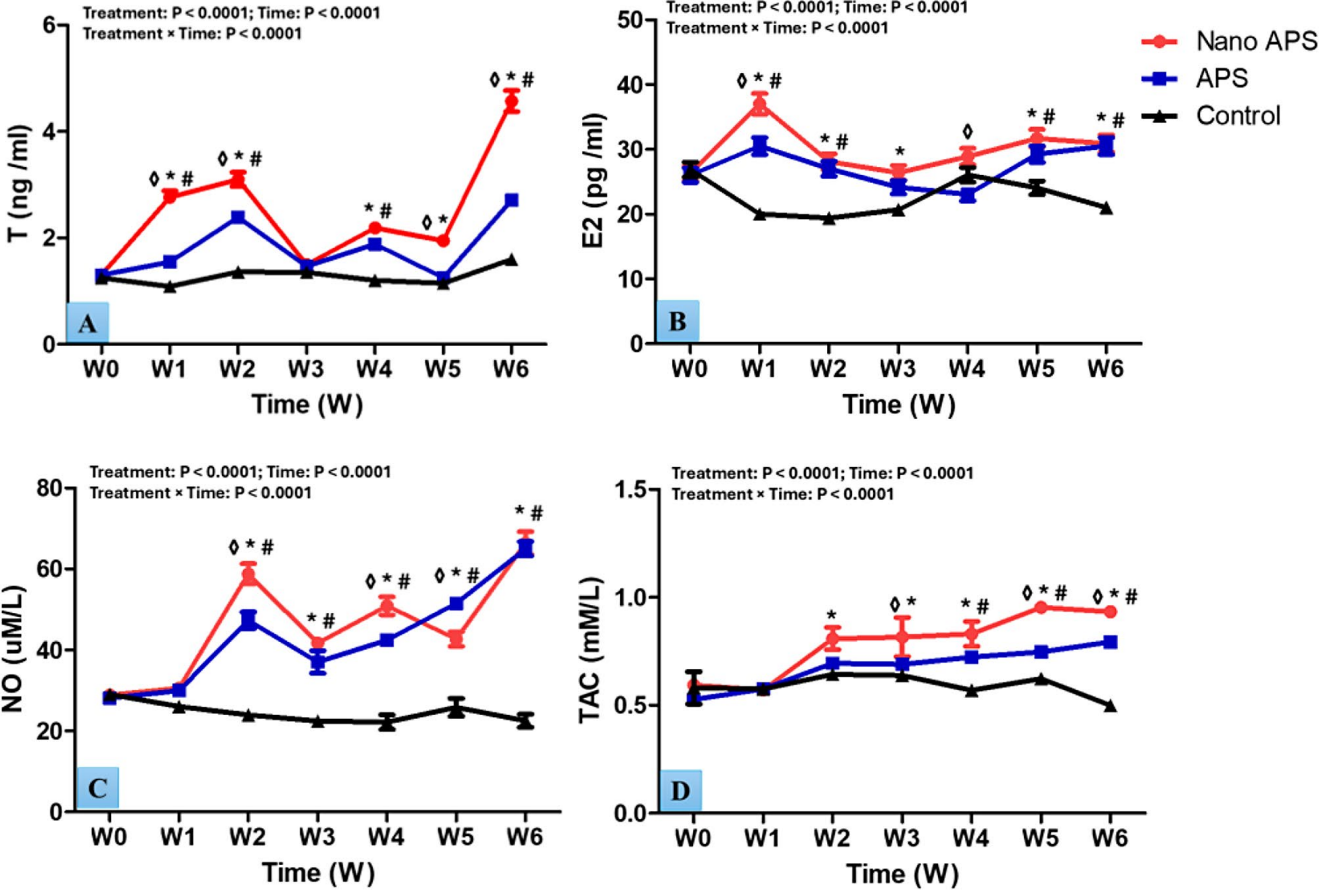
Changes in the concentrations of testosterone (T; ng/mL) (**A**), estradiol (E2; pg/mL) (**B**), nitric oxide (NO; µmol/L) (**C**), and total antioxidant capacity (TAC; Mm/L) (**D**) in Ossimi rams in the nano APS group, APS group, and the control group (*n* = 5, each) during different times (weeks). Values are means ± SEM. ◊ Values represent significant differences (*P* < 0.05) between nano APS and APS groups at the indicated times during the study. * Values represent significant (*P* < 0.05) differences between nano APS and control groups during the study. # Values represent significant (*P* < 0.05) differences between APS and control groups during the study

The serum levels of T (ng/ml), E_2_ (pg/ml), NO (µmol/L), and TAC ( mM/L) in the studied groups are shown in Fig. 8. In general, treatment, time, and interaction had significant differences (*P* < 0.0001, for all) in T, E_2_, NO, and TAC values. Concentrations of T were higher (*P* < 0.001) in the nano APS group at most time points of the study, compared to the APS one. Rams in the nano APS and APS groups attained higher (*P* < 0.001) concentrations of T at most time points of the study (W1, W2, W4, W5, and W6) and (W1, W2, W4, and W6), respectively, compared to that in the control one. E_2_ concentrations were higher (*P* < 0.01) in the nano APS group at W1 and W4 than in the APS one. Rams in the nano APS and APS groups had higher (*P* < 0.05) concentrations of E_2_ at most weeks of the study (W1, W2, W3, W5, and W6) and (W1, W2, W5, and W6), respectively, than that in the control one. Regarding NO concentration, there were significant (*P* < 0.01) differences between the nano APS group and the APS one at W2, W4, and W5. Rams in nano APS and APS groups had higher (*P* < 0.001) NO concentrations, especially at the time point from W2 to W6. Increases in the concentrations of TAC (*P* < 0.05) were observed in the nano APS group compared to that in the APS one at W3, W5, and W6, and its levels were greater (*P* < 0.01) starting from W2, compared to that in the control group. Furthermore, increases (*P* < 0.05) were noticed in the concentrations of TAC at W4, W5, and W6 in the APS group compared to its values in the control one.

### Semen quality

**Fig. 9:**
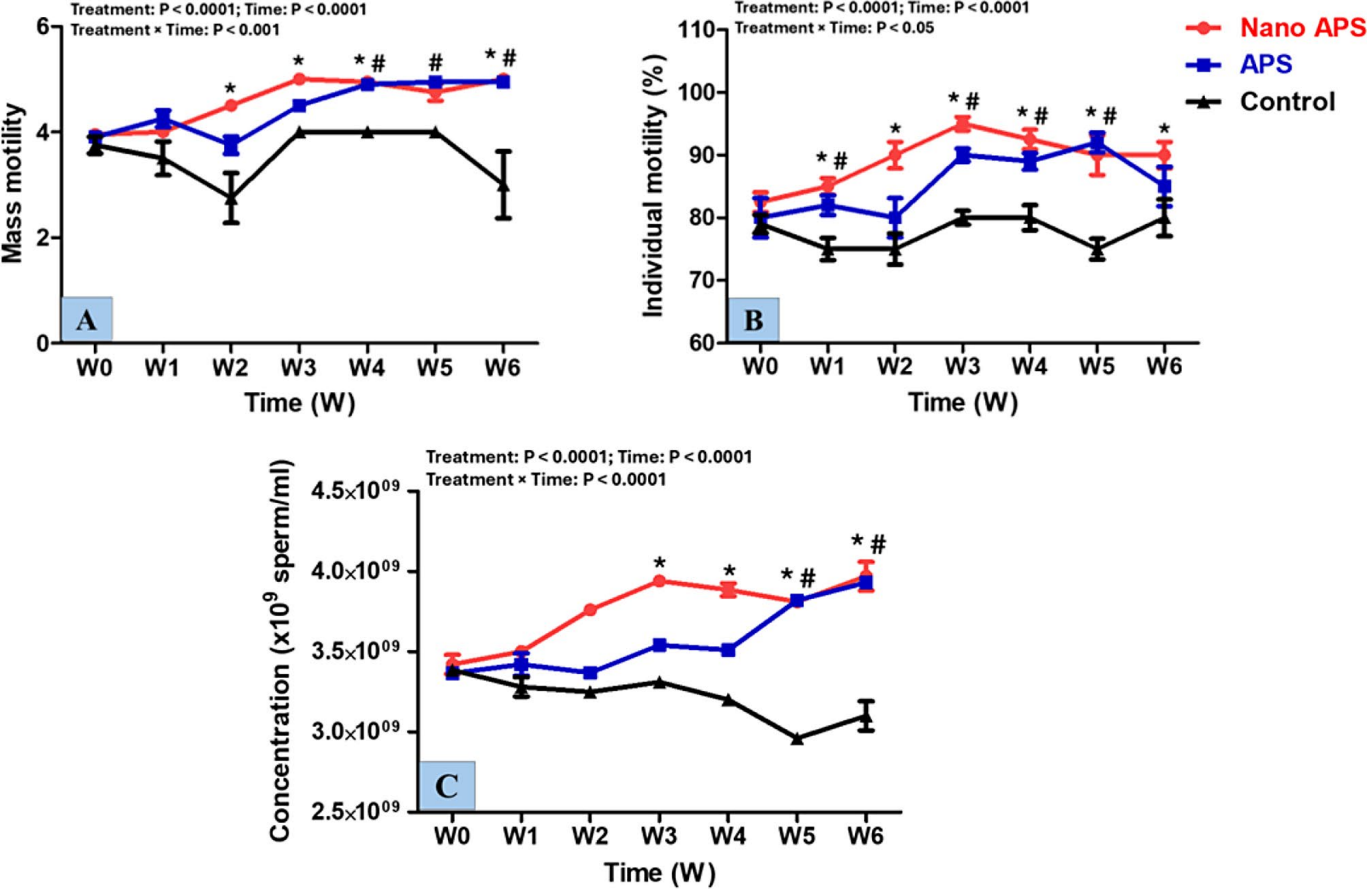
Semen characteristics [Mass motility (score 0–5) (**A**), individual motility (%) (**B**), and Sperm cell concentrations (10^9^ /ml) (**C**)] in the nano APS group, APS group, and the control group (*n* = 5, each) in Ossimi rams during different times (weeks). Values are means ± SEM. ◊ Values represent significant differences (*P* < 0.05) between nano APS and APS groups at the indicated times during the study. * Values represent significant (*P* < 0.05) differences between nano APS and control groups during the study. # Values represent significant (*P* < 0.05) differences between APS and control groups during the study

The results of mass motility, individual motility, and sperm cell concentration in the present study are summarized in Fig. 9. For the mass motility, there were treatment, time, and interaction (*P* < 0.0001, *P* < 0.0001, and *P* < 0.001; respectively) effects. In the nano APS, mass motility increased (*P* < 0.01) at most time points (W2, W3, W4, and W6) of the study, compared to the control one. Mass motility was higher (*P* < 0.01) in the APS group at W4, W5, and W6 compared to the control group. The individual motility (%) of rams’ spermatozoa had treatment, time, and interaction effects (*P* < 0.0001, for all). It was improved (*P* < 0.001) in the nano APS group at W2 compared to that in the APS one, and its levels were greater (*P* < 0.001) starting from W1 and onward compared to that in the control one. Furthermore, increases (*P* < 0.01) were noticed in the progressive motility % of spermatozoa in the APS group starting from W1 to W6 of the study, compared to that in the control one. For sperm cell concentration (x10^9^ sperm/ml), there were treatment, time, and interactions (*P* < 0.001, for all) effects. The sperm cell concentration had no significant (*P* > 0.05) differences during all study weeks (W0 - W6) between the nano APS and APS groups. Rams in the nano APS group had significantly higher sperm cell concentration from W3 to W6 (*P* < 0.01) than the control one and the APS group attained higher (*P* < 0.01) sperm cell concentration at W5 and W6 compared to the control one.

**Fig. 10:**
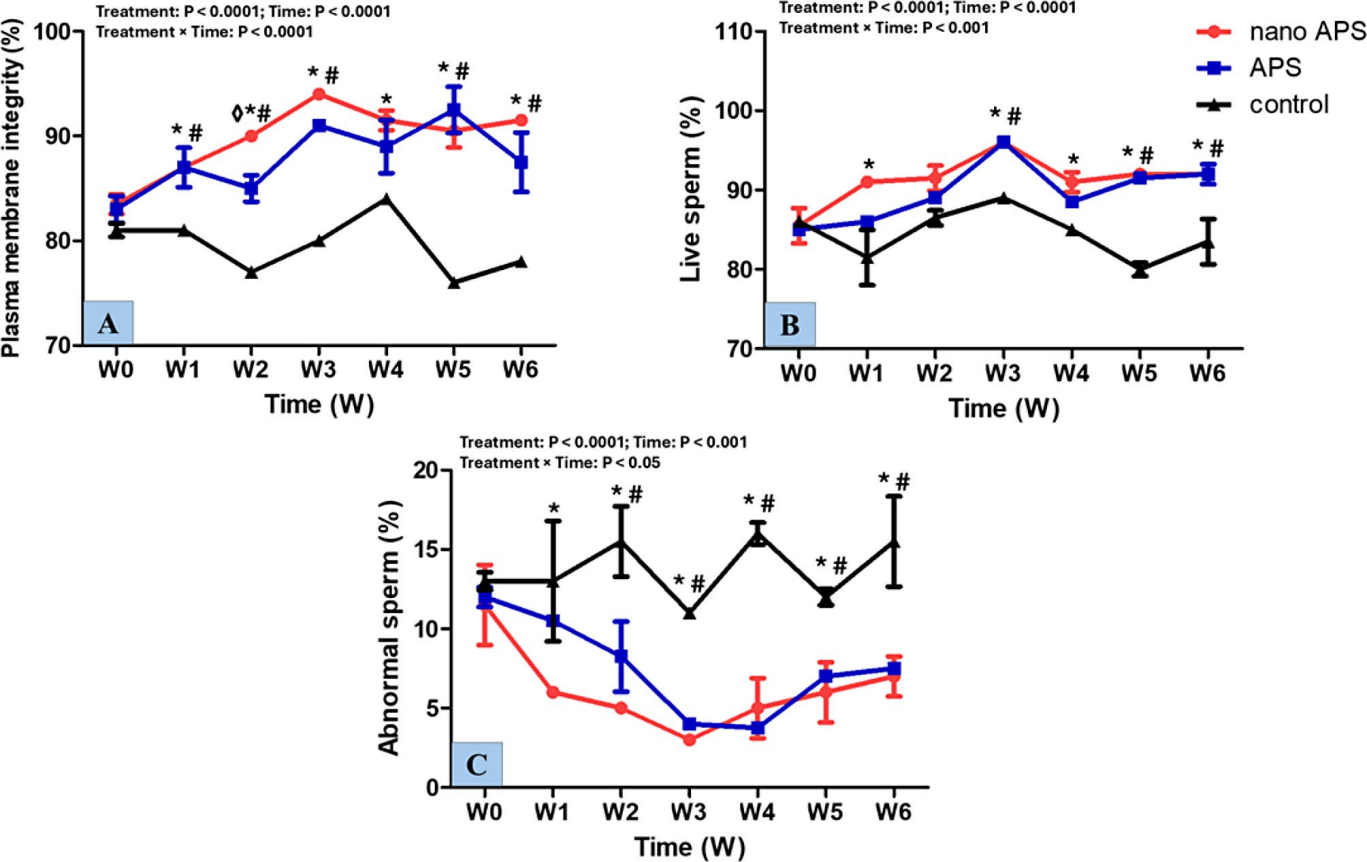
Semen characteristics [Plasma membrane integrity (%) (**A**), live sperm (%) (**B**), and abnormal sperm (%) (**C**)] in the nano APS group, APS group, and the control group (*n* = 5, each) in Ossimi rams during different times (weeks). Values are means ± SEM. ◊ Values represent significant differences (*P* < 0.05) between nano APS and APS groups at the indicated times during the study. * Values represent significant (*P* < 0.05) differences between nano APS and control groups during the study. # Values represent significant (*P* < 0.05) differences between APS and control groups during the study

As shown in Fig. 10, sperm plasma membrane integrity (%) had treatment, time, and interaction effects (*P* < 0.0001, for all). Rams’ spermatozoa in the nano APS group had a higher (*P* < 0.05) percentage of plasma membrane integrity than the APS group at W2. In nano APS and APS groups, plasma membrane integrity % was higher (*P* < 0.05) at time points from W1 to W6 compared to the control one. Treatment, time, and interaction were significantly different (*P* < 0.0001, *P* < 0.0001, and *P* < 0.001; respectively) in live sperm %. In the nano APS group live sperm % increased (*P* < 0.05) starting from W2 to W6 compared to the control one, and the APS group attained higher (*P* < 0.01) live sperm % at W3, W5, and W6 than the control. For abnormal sperm (%) of ram semen, treatment, time, and interaction were significantly different (*P* < 0.0001, *P* < 0.001, and *P* < 0.05; respectively). Abnormal sperm % was lower (*P* < 0.05) in the nano APS group starting from W1 to W6 compared to the control, moreover, it decreased (*P* < 0.05) in the APS group from W2 to W6 compared to the control.

## Discussion

For thousands of years, *Astragalus membranaceus* has been utilized in traditional Chinese medicine to boost innate immunity. The primary active component of *Astragalus membranaceus* is APS. Numerous biological activities of APS have been demonstrated, including immunomodulation [51, 52], antioxidant [53, 54], anti-inflammation [55, 56], antitumor [57], anti-diabetes [58], antiviral [59], hepatoprotection [60, 61], anti-atherosclerosis [62], hematopoiesis [63], and neuroprotection [64]. Despite multiple studies supporting *Astragalus membranaceus’* potential medical benefit, its effects on the reproductive system have received little attention. The present study investigated, for the first time according to the best of the authors’ update, the influence of APS and nano APS on testicular hemodynamics and semen quality in Ossimi rams. The results of the current study supported our hypothesis that the dietary administration of APS and nano APS significantly influenced testicular hemodynamics and improved semen quality in Ossimi rams. The provision of such data is crucial for the continued improvement of animal productivity as well as a tool for helping to resolve various issues related to ram fertility in the future.

In the current investigation, the testicular artery Doppler indices (RI, PI, and S/D) significantly decreased in response to APS and nano APS treatment. These indices show the blood flow resistance in the vessels under examination, which in turn indicates the flow conditions downstream [65, 66]. Blood flow resistance dropped when the values dropped and vice versa [10]. Reduced RI and PI values are thought to be the consequence of increased blood flow, and these variations in values are significant if the corresponding organ requires a constant supply of oxygen and nutrients [67]. Although the exact mechanism by which APS increased TBF was not made clear in this investigation, several approaches potentially explain these mechanisms. First, APS could improve vascular health through its effects on NO status [68]. The present study showed that APS and nano APS administration significantly increased the NO concentrations. Prior research has suggested that therapy with *Astragalus membranaceus* improves endothelial function by increasing NO and cyclic guanosine monophosphate (cGMP) synthesis in the heart and aorta [69]. In other words, *Astragalus membranaceus* may enhance endothelium-dependent vascular relaxation through its modulation of the NO/cGMP system. Notably, NO is synthesized from L-arginine methyl ester amino acid by NO synthase enzyme in different peripheral tissues, involving the testicular vasculature and seminiferous tubules [70]. Nitric oxide is produced in the vascular endothelial cells and is rapidly inactivated by reactive oxygen species (ROS) such as superoxide anion radical (O_2_) to produce peroxynitrite [71]. Therefore, the authors suggested the stimulatory effect of APS on TBF that was observed in the present study may be due to elevated levels of NO which is considered one of the powerful vasodilators [72, 73]. Furthermore, earlier research demonstrated the crucial roles of NO in controlling testicular hemodynamics and basal blood vessel tone [72, 74]. Second, APS enhances microcirculation and red blood cell fluidity as reported in cases of cerebral thrombosis [75] with APS nanoparticles having a stronger effect than APS as reported in the rats model [76]. In the present study, the nano APS group had lower Doppler indices values than the APS group; this might be due to the nano APS’s tiny size and ability to pass through the blood-testis barrier [77–81]. Furthermore, numerous studies revealed that the coating of the nano-carrier increased the specific surface area of the Chinese medicine’s active ingredient, extending the drug’s in vivo retention period and offering the benefits of sustained release and targeting [82, 83].

Testosterone and E_2_ concentrations in the treatment groups were higher than those in the control group. According to prior research on rat Leydig cells produced in vitro, one possible method by which APS upregulates T and E_2_ secretion is by its direct action on the testis, where it increases the number of Leydig cells [84]. Measuring E_2_ is useful in understanding its involvement in the reproductive function of rams because it has a crucial role in the regulation of the hypothalamic-pituitary-gonadal axis; gonadotropin secretion (LH and FSH), testosterone synthesis, and spermatogenesis. Additionally, E_2_ plays critical functions in controlling TBF due to its strong vasodilatory impact [10, 85]. Several investigations conducted on goats [86], rams [87], and stallions [10] found a strong correlation between E_2_ levels and Doppler indices of blood flow in the testicular artery. Estradiol also has a role in regulating the blood flow to the uterus and ovaries in females, and its exogenous treatment enhances the blood perfusion of these organs [88]. The underlying mechanism for the vasodilatory effect of E_2_ on testicular arteries has not been fully elucidated, but its vasodilatory effect on uterine blood flow may be mediated by intracellular signaling, which involves reductions in the calcium uptake of potential sensory channels by the E_2_ receptors in the tunica media of the uterine arteries [89].

The study of testicular echogenicity provides an additional noninvasive testicular function evaluation by PIX estimation [41]. In the current study, reductions in PIX and IND values following APS and nano APS treatment may be attributed to increased blood perfusion of the testis. Increased blood perfusion (due to lower TBF RI and PI values) may generate an increase in intratesticular fluids, which reduces echotexture PIX. Our assumption is consistent with prior findings in rams [87, 90] and bucks [91]. In the present study, the testicular volume and scrotal circumference did not alter significantly after the administration of APS and nano APS since testicular dimensions are more closely associated with growing rams, while rams in the current study were sexually mature.

During most time points in the current study, the APS and nano APS groups demonstrated significant increases in sperm quality parameters, such as mass motility, progressive motility percent, spermatozoa concentration, alive sperm percent, and decreased abnormal spermatozoa, with nano APS outperformed APS in this regard. These enhancements may be explained by the significant systemic or local functions that APS plays in the testis such as boosting testicular enzymatic activities of acid phosphatase (ACP), sorbitol dehydrogenase (SDH), and lactate dehydrogenase (LDH), resulting in improved testicular functioning in breeder cocks [92], as well as by increases in TBF, which may act as a trigger for the testis’s endocrine activity. Steroidogenesis and spermatogenesis, two endocrine and exocrine testicular activities, were found to be positively correlated with reduced RI and PI [11, 93]. Furthermore, testicular RI and PI indices are strongly related to sperm quality metrics in stallions [13] and the spermatogenesis process in dogs [94] and humans [4]. These findings may be attributable to APS conserving spermatozoa mitochondria and boosting energy metabolism, as described in frozen bull semen [21].

According to some research, APS can shield cells by enhancing their viability, lowering apoptosis, and preventing the release of inflammatory cytokines [95]. Furthermore, it has an antioxidant impact, as demonstrated in the current study, by boosting TAC concentration. It is well known that APS has strong ROS-scavenging abilities as well as a chelating impact on ferrous ions in vitro [96]. The available literature on APS in domestic animals, in the field of reproduction, is limited. As such, the antioxidant activity of APS has been demonstrated in various animal studies. For weaned lambs, administering APS at a dosage of 15 g/kg in meals for 30 days might dramatically boost plasma TAC levels [97]. Additionally, APS also enhanced the antioxidant capacity and sperm quality of frozen-thawed goat spermatozoa [19]. For mice, dietary APS supplementation (160 mg/kg body weight) for 30 days increased antioxidant enzyme activity, while it decreased MDA levels in the blood and liver [98].

Previous literature that investigated the effect of ASP or its nanoparticles on the fertility potentials of farm animals was scarce and limited to in vitro studies or cryopreservation of the semen. This study is the first that endorsed the efficacy of APS and its nanoparticles on the testicular hemodynamics, echotexture, and semen quality in rams. Depending on the findings of this study, the dietary administration of both APS and nano APS improved the examined parameters compared to the control group. However, the nano APS had more potent effects in improving most studied parameters during the study. Moreover, in the nano APS group, we used a lower dose (2 g/ram/day) compared to the APS group (20 g/ram/day). Understanding the mechanistic action may be out of the scope of this study. Astragalus polysaccharides lack significant activity because it is difficult to penetrate cell membranes or some of them are absorbed from the cell interstitial space. With the advantages of nanoparticles such as strong cell penetration and targeting ability, effective biodistribution and pharmacokinetics, and good biocompatibility, the construction of APS nanopreparations can improve its absorption efficiency and increase its concentration at the target site, so its effect can be high. The results show that under the given synthesis conditions, the prepared nano-APS exhibited a zeta potential of + 31.2 ± 0.01 mV, indicating that positively charged groups surround the nanoparticles and provide stability. The average particle size was measured to be 59.10 ± 0.20 nm as shown in Fig. 1. This result indicates that the simple aqueous extract of *Astragalus membranaceus* effectively produced nano-APS with reduced particle size and increased stability, probably due to the increased zeta potential. Furthermore, the presence of a single peak indicates that the quality of the synthesized nano-APS is high. One of the important purposes of nanoparticles is to prolong their residence time in the biological system and thus indirectly increase their strength. Positively charged nano-APS are absorbed faster than cell membranes if they are negatively charged. However, the absorption of positively charged synthesized nano-APS also carries the risk of causing toxicity and cell death by destabilizing membrane integrity (causing cell fluidity). Ultrasound findings and sperm quality results may indicate that nano-APS does not have a toxic effect. The small size of nano-APS may reduce the rate of elimination from tissues. The high zeta potential value of nano-APS, possibly low dissolution kinetics and physicochemical stability increased sperm quality until the 3rd week of the experiment and maintained it until the 6th week of the experiment. The antioxidant activity measured was high, consistent with the results of previous studies. Thus, the authors think that the administration of the nano APS is superior to the APS for enhancing the reproductive performance in rams based on the examined parameters.

This study has provided important insights into the positive effects of the dietary supplementation of APS in rams. These results are a good step forward. Since, there were limitations in this study, future studies on a larger scale would be required. Where working with a larger number of animals allows for more exact dose calculation and standardized dosage per kilogram of body weight rather than per animal. Furthermore, allowing to examine fertilizing capacity (pregnancy outcomes) which will help assess the practical outcomes of these findings on reproduction, eventually opening the way for larger applications in the field.

## Conclusion

The dietary administration of APS and nano APS can enhance the TBF as measured by pulsed-wave Doppler ultrasonography (by decreasing values of RI, PI, and S/D of testicular arteries). Concurrently, it hastened sperm characteristics, testicular echotexture, and the concentration of serum T, E_2_, NO, and TAC with a more significant effect and lower dose in the APS nanoparticles compared with APS. Thus, this study shows the practical potential of using nano APS as a dietary supplement (2 g/ram/day) for four weeks to improve the reproductive performance of rams.

## Electronic supplementary material

Below is the link to the electronic supplementary material.

Supplementary Material 1

## Data Availability

The data that support this study are available in the article and a supplement file 1.
