# Supplementary material for: Dietary supplementation of Astragalus polysaccharide or its nanoparticles enhances testicular hemodynamics, echotexture, scrotal circumference, concentration of testosterone, estradiol, nitric oxide, and total antioxidant capacity, and semen quality in mature Ossimi rams
Source: BMC Vet Res. 2025 Feb 6;21:55. doi: 10.1186/s12917-025-04477-6 (PMC11804079; doi:10.1186/s12917-025-04477-6)
Supplement: Supplementary file 1 — Supplementary Material 1 [file 12917_2025_4477_MOESM1_ESM.docx]

**Supplement Table 1: Feed ingredients and composition**

| **Feed ingredients (%)** | |
| --- | --- |
| Soybean meal | 5 |
| Cotton seed cake | 15 |
| Wheat bran | 25 |
| Yellow corn | 52 |
| Ground limestone | 1.7 |
| Common salt | 0.8 |
| Mineral-vitamin premix | 0.5 |
| **Feed composition (%)** | |
| Crude protein | 14 |
| Ether extract | 4 |
| Total digestible nutrients | 68 |
| Fiber | 8 |
| Ash | 6 |

Mineral and vitamin premix is composed of phosphorus, magnesium, potassium, sulphur, chlorine, vitamin B complex, selenium, and yeast.
